# Determination of Polycyclic Aromatic Hydrocarbons (PAHs) in Leaf and Bark Samples of *Sambucus nigra* Using High-Performance Liquid Chromatography (HPLC)

**DOI:** 10.3390/mps6010017

**Published:** 2023-02-08

**Authors:** Fausto Viteri, Nazly E. Sánchez, Katiuska Alexandrino

**Affiliations:** 1 Grupo de Protección Ambiental (GPA), Facultad de Ciencias de la Ingeniería e Industrias, Universidad UTE, Quito 170527, Ecuador; 2 Departamento de Ingeniería Ambiental y Sanitaria, Universidad del Cauca, Popayan 190007, Colombia; 3 Facultad de Ingeniería y Ciencias Aplicadas, Ingeniería Agroindustrial, Universidad de Las Américas, Quito 170503, Ecuador

**Keywords:** PAH, biomonitoring, air pollution, tree, HPLC

## Abstract

Polycyclic aromatic hydrocarbons (PAHs) are ubiquitous organic compounds coming from natural or anthropogenic activities. Tree organs such as leaves and barks have been used to monitor urban air quality and have achieved remarkable ecological importance. However, the potential of many tree species as biomonitors is still unknown and efforts should be focused on conducting studies that analyze their capabilities with a viable analytical method. In this work, an analytical method for quantification of the 16 EPA priority PAHs from the leaves and bark of *Sambucus nigra* was validated. In general, the method showed good linearity, detection limits, precision, and recoveries, demonstrating that it is suitable for analyzing PAHs in both the leaves and bark of the *Sambucus nigra* species for which no analytical method for PAHs is yet available. The high prevalence of fluoranthene in the samples, which is a PAH related to coal combustion and biomass burning, and benzo[a]pyrene, which has a carcinogenic effect, was identified.

## 1. Introduction

Polycyclic aromatic hydrocarbons (PAHs) are a series of organic compounds containing two or more fused benzene rings that form during the incomplete combustion of organic matter [1,2]. Their emissions may be due to natural or anthropogenic activities [3]. Approximately 500 different PAHs have been detected in the air [4]. However, only 16 PAHs have been classified by the United States Environmental Protection Agency (U.S. EPA) as priority pollutants due to their high carcinogenic and mutagenic potential [5]. These are: naphthalene (Naph), acenaphthylene (Acy), acenaphthene (Ace), fluorene (Fluo), phenanthrene (Phen), anthracene (Ant), fluoranthene (Flt), pyrene (Pyr), benzo[a]anthracene (BaA), chrysene (Chry), benzo[b]fluoranthene (BbF), benzo[k]fluoranthene (BkF), benzo[a]pyrene (BaP), dibenzo[a,h]anthracene (DahA), benzo[g,h,i]perylene (BghiP), and indeno [1,2,3-cd]pyrene (IcdP).

PAHs exist in the atmosphere in the vapor and/or in particle-bound phase, and a large portion of them are scavenged by vegetation via dry and wet deposition [6]. In this sense, the use of vegetation, especially trees, in the assessment of atmospheric PAHs’ concentrations has gained great interest due to its low cost. Moreover, due to their high spatial and temporal distribution, the use of trees provides the possibility of building high-resolution maps of air pollution to detect risk areas in urban areas. However, differences in the ability to accumulate PAHs between tree species have been identified [7,8,9].

The interception of pollutants by trees take place mainly in the upper portion of the tree, such as leaves, stems, and barks. In this sense, different works have addressed the use of leaves/needles from different tree species to assess the presence of PAHs in urban environments [7,8,9]. Stomata and outer cuticular lamellae are main vias for the uptake of PAHs in the vapor phase, whereas particle-bound PAHs are accumulated on the leaf surface [9,10]. Other vegetative parts of the tree, such as bark, have been less studied, although some works have shown its good capacity to accumulate PAHs due to its high lipid content, and porous and almost inert surface [11,12,13].

The evaluation of the atmospheric PAH concentrations using the leaves and barks of different tree species is possible due to the development and application, in recent years, of some analytical procedures. The processes proposed in the literature vary, due to the complexity of the sample matrix. However, some steps in those protocols are similar, including sample pre-treatment, extraction, clean-up, pre-concentration, and chemical analysis. Furthermore, the ways of carrying out these processes are diverse.

Considering the sample pre-treatment, some studies include the use of drying techniques such as freeze drying [14], stoves [15], and ovens [16]. Moreover, crushing techniques using mortars [17], high-speed grinders [18], or liquid nitrogen [19] can also be used. However, there are many works where the intact samples are used, without any prior drying or crushing treatment [11,20,21,22]. The pre-treatment step has been shown to be a bottleneck in achieving adequate recoveries. Hence, it is important to pay attention to how the samples are prepared, as using severe methods can greatly reduce these recoveries [14].

Regarding PAHs extraction, ultrasonic extraction [11,18,20], Soxhlet extraction [18,21,23], accelerated solvent extraction [18,22,24], and microwave-assisted extraction [11,25] are the most used techniques, which involve the use of different organic solvents for better yields.

Extract clean-up, which is a step that is often necessary to remove some matrix co-extractant compounds, such as lipidic compounds and chlorophylls, which could cause interference and introduce errors in the analysis [26,27], is usually performed by column chromatography or solid-phase extraction (SPE) cartridges with different sorbents such as florisil [7,28], silica gel [13,21,29], or alumina [30,31]. Regarding pre-concentration, the rotary evaporator [17] and the nitrogen stream [32] are the common techniques used. For instrumental analysis, gas chromatography coupled to mass spectrometry (GC-MS) is the most widely used equipment for the detection and quantification of PAHs [14,22,23,24,25,33]. High-performance liquid chromatography with diode array (HPLC-DAD) and/or fluorescence detectors (HPLC-Fl) is another technique used, although to a lesser extent than GC-MS [20,21,32].

Although several analytical methods for the identification and quantification of PAHs have been developed in recent years, a method developed for one tree species may not be suitable for other ones. It may not even be suitable for another vegetative part of the same tree. Therefore, the development of accurate and sensitive analytical methods is necessary for the determination of PAHs in different tree species and their vegetative parts. To the best of our knowledge, no study has been carried out for the leaves and bark of *Sambucus nigra*. This is a deciduous multi-stemmed small tree native to Europe, southwestern Asia, and northern Africa, and introduced and widely dispersed in Ecuador and South America in general. This tree species has different medicinal and food uses. On the one hand, their flowers and fruits have flavonoids, organic acids, essential oils, phenolic acids, and anthocyanins showing an antiviral effect, strengthening the immune system and providing inmuno-protection [34]. Moreover, their leaves, berries, and flowers seem to act as antioxidants by neutralizing free radicals [35]. On the other hand, the fruit provides flavor and color to certain foods, and it is used to prepare preserves, wines [36], sponge cakes [37], among other foods.

*Sambucus nigra* can be found in pedestrian areas, parks, and main streets in urban and sub-urban areas, being useful for extensive spatio–temporal sampling; thus, its study as a biomonitor is interesting and necessary. Therefore, the aim of this work was to present an analytical method for the quantitative extraction and determination of 16 US-EPA PAHs in leaf and bark samples of *Sambucus nigra*. The analytical procedure includes the use of an ultrasonic bath for extraction, concentration, an SPE clean-up procedure, and the final concentration before analysis by high-performance liquid chromatography (HPLC).

## 2. Materials and Methods

### 2.1. Sample Collection

Leaf and bark samples of *Sambucus nigra* were collected in a residential area (0°10′38.5″ S 78°21′51.1″ W) in the city of Quito, Ecuador. Sampling was performed from all directions of the tree at a specific height and using a new pair of powder-free vinyl gloves for each sample to avoid cross contamination. Specifically, eight branches were collected from the outer part of the tree by using a pruning shear and at a height of approximately 2 m above the ground. On the other hand, the bark was carefully removed from the boles of the tree at a height of approximately 1.5 m above the ground using a steel knife. Between each sampling, the pruning shear and the steel knife were cleaned with alcohol.

All the collected branches and barks were packed together in a single Ziplock bag, respectively. The bags were then labeled on site with the name of the species tree, sample type (leaf or bark), the date of collection, and GPS coordinates. To avoid photochemical degradation and volatilization of PAHs, bags were wrapped in aluminum to protect them from light and placed in cooler containing ice packs. Finally, samples were transported to the laboratory and stored at −20 °C for four days, for subsequent sample treatment and chemical analysis.

### 2.2. Sample Treatment

Prior to extraction, the samples were defrosted in a desiccator. Then, 2 g of leaves, of identical length and with no evidence of chlorosis or necrosis, were randomly taken by hand from the branches, taking care to minimize contact with the leaf surface, and weighed in six 250 mL beakers. Likewise, 2 g of bark, without the presence of mold, fungi, lichens, or foreign material such as spider webs, was weighed in six 250 mL beakers. Powder-free vinyl gloves were used to avoid cross contamination (a new pair between each weighing).

To evaluate the performance of the method (%Recovery (%R)), 0.3 mL of a 10 μg mL^−1^ certified standard mixture of 16 EPA PAHs in acetonitrile (SigmaAldrich, purchased from Supelco, Ecuador) was added into three of the six beakers with leaves and barks, respectively (final concentration of 1.5 μg g^−1^). This allows determining the recoveries by the matrix spike method in triplicate, which is a widely used procedure for evaluating the performance of a method in the absence of a reference material [38,39,40].

### 2.3. PAH Extraction

For the extraction procedure to recover the target analytes, an ultrasonic bath was used. This equipment is normally available in laboratories and has been used in different research works to extract PAHs from plant material [11,18,20].

An amount of 20 mL of a dichloromethane/hexane (1:1 *v*/*v*) mixture was added to each of the beakers (with the spiked and non-spiked sample) prepared in the previous step. The tops of the beakers were covered with aluminum foil and immersed in a 420-W ultrasonic bath for 10 min. This procedure was repeated two more times, using the fresh solvent mixture, for a total of 30 min of extraction and 60 mL of dichloromethane/hexane mixture for each sample. The three extracts of each sample were combined in a round bottom flask (100 mL) and evaporated on a Buchi rotary evaporator at 30 °C, with a pressure between 550 mbar and 170 mbar, to approximately 1 mL, and further cleaned-up.

### 2.4. Clean-Up and Final Concentration

Sep-Pak Alumina cartridges (6 cc, 1 g. Waters) were used for cleaning-up. Firstly, the cartridges were placed in a Waters SPE Vacuum Manifold and conditioned by passing 10 mL of the dichloromethane/hexane mixture through the bed with a flow rate of approximately 1.4 drops per second obtained by adjusting the vacuum. The mixture was collected in a test tube and was discarded. Then, the column was loaded with the extract and 10 mL more of the dichloromethane/hexane mixture was added to allow the elution of the analytes with the same flow rate of 1.4 drops per second, which were collected in a clean test tube. Finally, 5 mL of dichloromethane was added. The eluted extract was transferred to a round bottom flask (100 mL) to evaporate it again to approximately 1 mL under the same conditions indicated above. After that, the extract was transferred to 2 mL Eppendorf and concentrated to dryness using a Genevac miVac centrifugal concentrator at 40 °C for 30 min. Finally, the samples were reconstituted in 1 mL of acetonitrile, shaken, filtered using a PVDF syringe filter (32 mm, 0.22 μm) attached to a syringe of 3 mL, and transferred to 2 mL amber glass vial. This final filtration is carried out before the chemical analysis to avoid the obstruction of the HPLC column due to the presence of any particle.

### 2.5. PAH Analysis

The samples were analyzed by a HPLC (Agilent 1260 system) using a ZORBAX Eclipse PAH column (4.6 × 50 nm, 3.5 µm) and a UV detector (Agilent 1260 DAD G4212B) operating with wavelengths (λ) of 220 nm, 230 nm, and 254 nm. The column temperature was maintained at 25 °C, the injection volume was set to 20 μL, and the flow rate was 1.4 mL/min. The elution program was defined as follows (with acetonitrile (A) and water (B) as mobile phases): 0–6 min isocratic 40:60 (*v*/*v*) A:B; 6–9.5 min linear gradient from 40 to 100% of A and 9.5–12 min isocratic 40:60 (*v*/*v*) A:B. The peak intensity of each PAH changes depending on the UV wavelength; thus, the PAHs were calibrated at the wavelength where the intensity was greatest for that PAH. Table 1 shows the retention time and the UV wavelength at which the peak of each PAH was most intense. The PAH peaks in the sample chromatograms were identified by a retention time matching between standard and sample chromatograms. Quantification was performed by the peak area of each PAH using the ChemStation software (Agilent Technologies, Santa Clara, CA, USA).

### 2.6. Method Validation

Linearity, limit of detection (LOD), limit of quantification (LOQ), repeatability, and recovery were determined for validation of the HPLC method.

External standard calibration curves were obtained using the certified standard at eleven different levels in the concentration range of 2.5–2500 μg L^−1^. The linearity of each PAH was evaluated as the coefficients of determination (*R*^2^) by regression analysis.

Instrumental LOD and LOQ were calculated according to Equations (1) and (2), respectively [14,41,42]:

(1) $$\mathrm{LOD}=\frac{3.3\sigma \;}{\mathrm{IC}}$$

(2) $$\mathrm{LOQ}=\frac{10\sigma \;}{\mathrm{IC}}$$

where IC is the calibration curve inclination and σ is the standard deviation of the intercept of the calibration curve with the *y*-axis.

Instrumental repeatability (precision) was studied as percent relative standard deviation (%RSD_inst_) of three consecutive injections of the standard solution at 100 μg L^−1^, while method repeatability was expressed as %RSD_method_ of the concentrations determined in duplicate spiked samples.

The recovery values (%R) were determined according to Equation (3) [43]:

(3) $$\%R=\frac{\mathrm{PAH}\;\mathrm{concentration}\;\mathrm{in}\;\mathrm{the}\;\mathrm{spiked}\;\mathrm{sample}-\;\mathrm{PAH}\;\mathrm{concentration}\;\mathrm{in}\;\mathrm{the}\;\mathrm{non}-\mathrm{spiked}\;\mathrm{sample}\;}{\mathrm{known}\;\mathrm{added}\;\mathrm{PAH}\;\mathrm{concentration}\;\mathrm{in}\;\mathrm{spiked}\;\mathrm{sample}}\times 100$$

## 3. Results and Discussion

### 3.1. Method Validation

**Figure 1:**
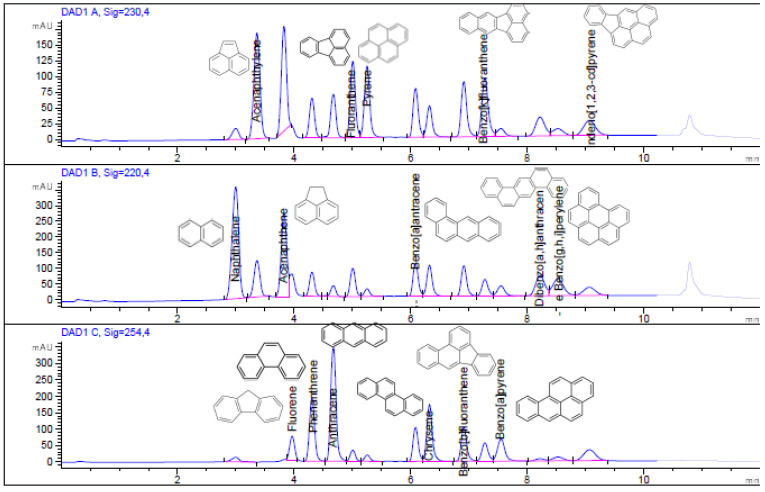
HPLC chromatogram of the certified standard at a concentration of 1000 μg L^−1^ of 16 PAHs at λ = 230 nm, 220 nm, and 254 nm. Each PAH was calibrated in the UV wavelength (λ) where their signal was greatest.

**Figure 2:**
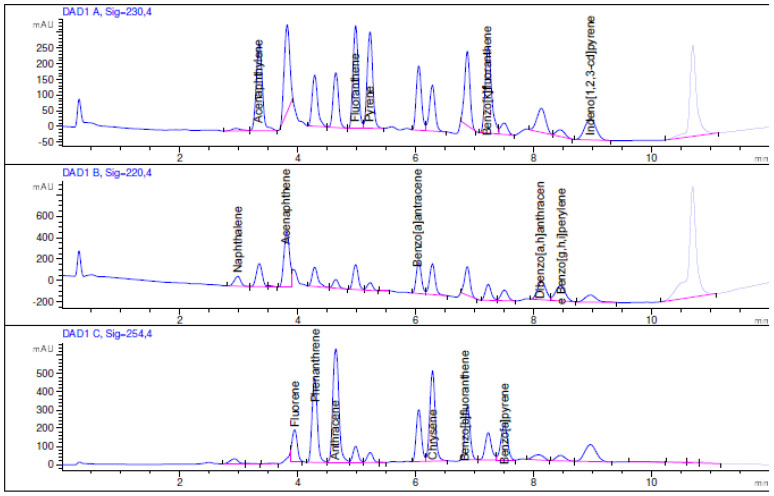
HPLC chromatogram of the spiked bark sample at 1.5 μg g^−1^ at λ = 230 nm, 220 nm, and 254 nm.

**Figure 3:**
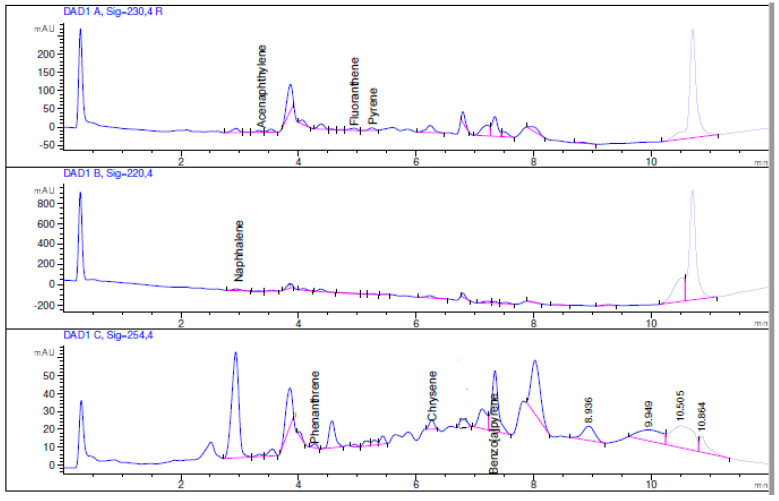
HPLC chromatogram of the non-spiked bark sample at λ = 230 nm, 220 nm, and 254 nm.

The concentration range of calibration and linearity for each PAH, the instrumental limit of detection (LOD) and quantification (LOQ), instrumental and method repeatability expressed in terms of percent relative standard deviation (%RSD), and recovery values are reported in Table 2 and Table 3. Moreover, as an example, Figure 1, Figure 2 and Figure 3 show the chromatogram of the certified standard at 1000 μg L^−1^, the spiked bark sample at 1.5 μg g^−1^, and the non-spiked bark sample.

All calibration curves show good linearity with R^2^ values ranging between 0.9993 and 0.9998 (Table 2). Instrumental LOD and LOQ ranged from 0.2 μg L^−1^ for Naph to 13.7 μg L^−1^ for IcdP, while LOQ values ranged from 0.6 μg L^−1^ for Naph to 41.5 μg L^−1^ for IcdP (Table 2). The instrumental repeatability values (*%RSD_inst_*) ranged between 0.006% and 4.6% (see Table 2), being within the interval values found in the literature (1–47.3%) [44,45] and indicating good instrumental precision.

In general, the method repeatability (%RSD_method_) was below 17% for the leaf and bark samples (Table 3), which is acceptable at such low concentration levels. An exception is observed for IcdP in both sample types and for BghiP in leaf samples. The high variability of high-molecular-weight PAHs has also been shown in previous works [46] and could be due to the remaining co-extracted interferences from leaves and bark. Moreover, the %RSD_method_ values were similar to those reported in the literature (up to 18.8% [8] and 31.4% [44]).

Regarding recovery, the lowest values were found for the lighter PAHs, mainly Naph, Acy, and Ace (Table 3). The low recovery of the lighter PAHs could be due to the fact that they are more likely to be lost during sample handling and treatment, mainly in the evaporation/concentration step, and because lighter PAHs can penetrate further into the leaf tissues [47], making their extraction more complex. However, most %R values are within the 60−120% range, which is accepted as valid [48].

### 3.2. Real Contaminated Samples

**Figure 4:**
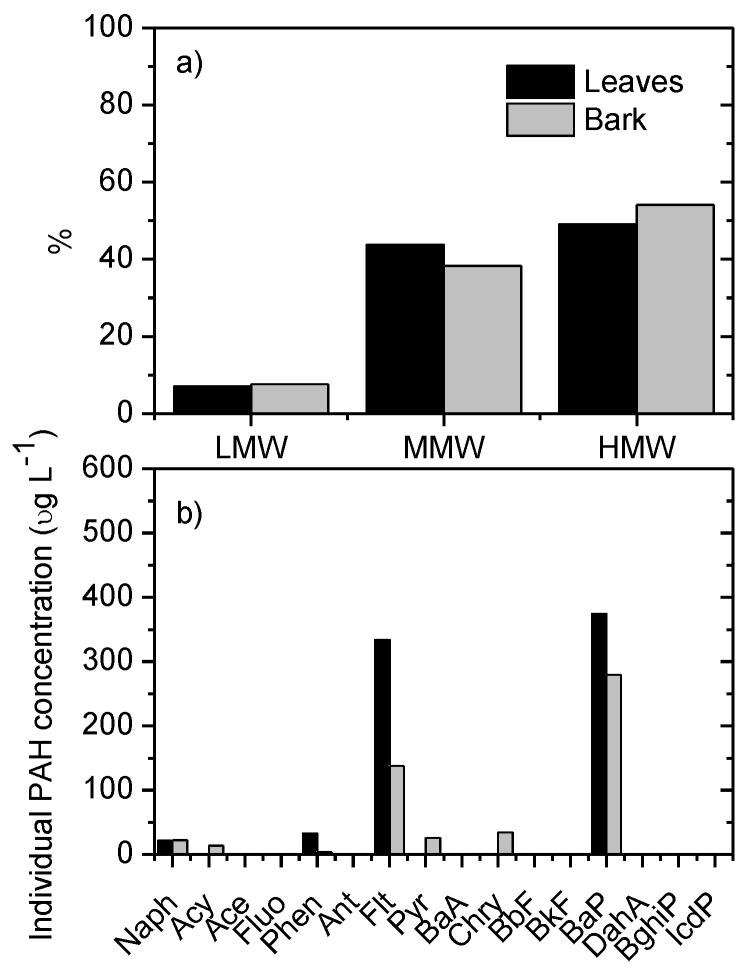
Experimental results for the non-spiked samples: (**a**) distribution of PAHs according to the molecular weight classification and (**b**) the individual PAH concentration.

Figure 4 shows the experimental data for the non-spiked leaf and bark samples, specifically: (a) the distribution of PAHs according to the molecular weight classification (light-molecular-weight PAHs (LMW: 2 and 3 rings PAHs), medium-molecular-height PAHs (MMW: 4 rings) and high-molecular-weight PAHs (HMW: 5 and 6 rings)); and (b) the individual PAH concentration. The concentration of each PAH was corrected based on the subtraction of the values of procedural blanks (extraction and clean-up of reagents without vegetative material) and the recoveries obtained in Table 3.

It is observed in Figure 4a that there is a greater predominance of HMW PAHs, followed by MMW, and finally LMW PAHs. The high incidence of HMW PAHs is due to the high concentrations of BaP (Figure 4b), which could indicate a high human exposure risk, as this PAH is the usual marker of carcinogenic levels of PAHs in environmental studies [49]. BaP is mainly attributed to gasoline exhaust emissions that are known to contribute to more BaP emissions than diesel engines [50]. Moreover, in a recent work [51], it was found that BaP is associated with acceleration and braking activities, i.e., with the presence of speed-modifying devices, such as traffic lights, roundabouts, intersections, curves, and speed bumps. On the other hand, the high incidence of MMW is attributed to the high concentrations of Flt (Figure 4b), which is associated with coal combustion and biomass burning [52]. The incidence of LMW PAHs was not as high and could be related to their high vapor pressures (higher volatility) which causes them to be resuspended into the atmosphere [8,30].

## 4. Conclusions

An analytical method for the detection and quantification of the 16 EPA priority PAH in leaf and bark samples of *Sambucus nigra* was validated. The methodology combines ultrasonic extraction, subsequent concentration and clean-up, final concentration, and the chemical analysis by high-performance liquid chromatography (HPLC). Linearity of the calibration curve, instrumental LOD and LOQ, instrumental and method repeatability (precision) (%RSD_inst_ and %RSD_method_), and recovery experiments were used to validate the method. Good precision was observed, obtaining instrumental repeatability in the interval of 0.006–4.6%, while most of the method repeatability was below 17%, with exception of IcdP in both sample types (32.8% for leaf and 45.8% for bark) and BghiP in leaf samples (24.0%). Most recovery values were within the accepted range of 60−120%. However, lower values were obtained for Naph, Acy, and Ace, indicating that there has been a loss of these analytes during sample handling and treatment, which probably occurred during the concentration stage. Results from the actual contaminated samples indicated a high incidence in the air of fluoranthene, which is associated with coal combustion and biomass burning, and of BaP, which is a PAH highly associated with gasoline exhaust emissions and has a carcinogenic effect.

## Figures and Tables

**Table 1:** Retention time and the UV wavelength (λ) at which the peak of each PAH is most intense.

	Retention Time (min)	λ (nm)
Naphthalene (Naph)	3.0	220.0
Acenaphthylene (Acy)	3.4	230.0
Acenaphthene (Ace)	3.8	220.0
Fluorene (FLuo)	4.0	254.0
Phenanthrene (Phen)	4.3	254.0
Anthracene (Ant)	4.7	254.0
Fluoranthene (Flt)	5.0	230.0
Pyrene (Pyr)	5.2	230.0
Benzo[a]anthracene (BaA)	6.1	220.0
Chrysene (Chry)	6.3	254.0
Benzo[b]fluoranthene (BbF)	6.9	254.0
Benzo[k]fluoranthene (BkF)	7.3	230.0
Benzo[a]pyrene (BaP)	7.5	254.0
Dibenzo[a,h]anthracene (DahA)	8.2	220.0
Benzo[g,h,i]perylene (BghiP)	8.5	220.0
Indeno [1,2,3-cd]pyrene (IcdP)	9.1	230.0

**Table 2:** Linear range, regression equation, coefficient of determination (R^2^), instrumental repeatability expressed in terms of percent relative standard deviation (%RSD_inst_), and instrumental limit of detection (LOD) and quantification (LOQ).

PAH	Linearity			%RSD_inst_	LOD (μg L^−1^)	LOQ (μg L^−1^)
	Concentration Range of Calibration (μg L^−1^)	Regression Equation *^a^*	R^2^			
Naphthalene (Naph)	7.5–2500	y = 2.4 ± 0.02x − 35.9 ± 21.53	0.9998	0.3	0.2	0.6
Acenaphthylene (Acy)	5.0–2500	y = 1.1 ± 0.008x − 8.2 ± 9.0	0.9997	1.7	0.8	2.5
Acenaphthene (Ace)	10–2500	y = 0.8 ± 0.008x − 6.8 ± 9.6	0.9996	0.2	2.6	7.8
Fluorene (FLuo)	10–2500	y = 0.2 ± 0.004x − 3.7 ± 4.63	0.9993	0.006	5.5	16.8
Phenanthrene (Phen)	5.0–2500	y = 0.9 ± 0.007x − 8.2 ± 7.14	0.9998	0.1	0.8	2.6
Anthracene (Ant)	2.5–2500	y = 1.9 ± 0.02x + 1.3 ± 9.0	0.9996	3.8	0.7	2.2
Fluoranthene (Flt)	5.0–2500	y = 0.6 ± 0.006x − 9.9 ± 6.4	0.9996	1.1	6.3	19.0
Pyrene (Pyr)	5.0–2500	y = 0.7 ± 0.005x − 7.6 ± 5.6	0.9997	0.3	7.8	23.8
Benzo[a]anthracene (BaA)	10–2500	y = 0.6 ± 0.006x + 0.4 ± 7.1	0.9996	1.0	1.6	4.9
Chrysene (Chry)	7.5–2500	y = 1.2 ± 0.01x − 14.5 ± 11.1	0.9997	0.5	1.4	4.2
Benzo[b]fluoranthene (BbF)	7.5–2500	y = 0.6 ± 0.006x − 5.3 ± 5.1	0.9996	4.6	4.8	14.7
Benzo[k]fluoranthene (BkF)	7.5–2500	y = 0.5 ± 0.004x − 6.8 ± 5.2	0.9996	0.4	6.3	19.0
Benzo[a]pyrene (BaP)	7.5–2500	y = 0.4 ± 0.004x − 1.4 ± 4.8	0.9995	4.3	1.2	3.7
Dibenzo[a,h]anthracene (DahA)	7.5–2500	y = 0.6 ± 0.005x − 6.4 ± 5.8	0.9997	0.9	6.1	18.4
Benzo[g,h,i]perylene (BghiP)	25.0–2500	y = 0.7 ± 0.008x − 14.4 ± 9.3	0.9997	1.2	6.5	19.6
Indeno[1,2,3-cd]pyrene (IcdP)	25.0–2500	y = 0.4 ± 0.003x − 9.2 ± 3.8	0.9997	0.4	13.7	41.5

^a^ Calibration curves constructed by linear regression of the peak area (y) of each PAH against their respective concentrations (x) (μg L^−1^).

**Table 3:** Percentage recoveries (%R) and method repeatability expressed in terms of percent relative standard deviation (%RSD_method_).

PAH	Leaves		Bark	
	%R	%RSD_method_	%R	%RSD_method_
Naphthalene (Naph)	74.8	3.7	56.7	6.3
Acenaphthylene (Acy)	64.8	1.3	58.6	5.5
Acenaphthene (Ace)	67.9	3.9	55.2	12.8
Fluorene (FLuo)	75.1	1.9	72.1	13.8
Phenanthrene (Phen)	106.4	2.3	100.6	4.5
Anthracene (Ant)	88.9	1.7	92.4	3.1
Fluoranthene (Flt)	90.2	3.3	79.9	1.0
Pyrene (Pyr)	77.4	1.7	69.8	3.2
Benzo[a]anthracene (BaA)	85.1	1.2	86.7	2.9
Chrysene (Chry)	95.7	2.7	82.1	5.0
Benzo[b]fluoranthene (BbF)	72.1	3.1	75.7	1.6
Benzo[k]fluoranthene (BkF)	70.0	7.6	73.2	5.8
Benzo[a]pyrene (BaP)	91.2	16.9	69.3	11.0
Dibenzo[a,h]anthracene (DahA)	83.9	5.1	74.7	10.1
Benzo[g,h,i]perylene (BghiP)	76.4	24.0	82.8	10.5
Indeno [1,2,3-cd]pyrene (IcdP)	75.7	32.8	64.3	45.8

## Data Availability

Data are available from the corresponding author on request.
